# Learn, Engage, and Act to Advance Age Inclusivity in Higher Education

**DOI:** 10.1093/geroni/igab046.861

**Published:** 2021-12-17

**Authors:** Joann Montepare

**Affiliations:** Lasell University, Newton, Massachusetts, United States

## Abstract

Shifting age demographics are reshaping our social structures with far-reaching implications for higher education. Aging populations mean more older adults are looking to higher education to meet their professional needs and personal interests, and the longevity economy is calling for a trained workforce to provide services to support the health and functioning of individuals as they age. As well, there is a need to improve students’ aging literacy, along with developing synergistic age-friendly campus-community partnerships to address aging issues. How can institutions explore, create, develop, and sustain more age-friendly programs, practices, and partnerships? This presentation will introduce the toolkit specially designed by the GSA-AGHE Workgroup for use by faculty, students, administrators, and other campus leaders, and will provide an overview of the Age-Friendly University (AFU) initiative and its 10 guiding principles for creating more age-inclusive campuses.

